# Association Between Various Metrics of the Rural Food Environment and Diabetes and Obesity

**DOI:** 10.5888/pcd23.260106

**Published:** 2026-09-24

**Authors:** Jessica Gjonaj, Haeseung Yi, Tammy A. Flores, Crystal So, Haley L. Motola, Demba Keita, Jessie Moore, Brian Elbel, Lorna E. Thorpe, David C. Lee

**Affiliations:** 1 Institute for Excellence in Health Equity, NYU Grossman School of Medicine, New York, New York; 2 Department of Emergency Medicine, NYU Grossman School of Medicine, New York, New York; 3 Sullivan County Public Health Department, Liberty, New York; 4 Department of Population Health, NYU Grossman School of Medicine, New York, New York; 5 Wagner Graduate School of Public Service, New York University, New York, New York

## Abstract

**Introduction:**

Associations between unhealthy food environments and increased risk of diabetes or obesity have been studied extensively in urban areas but less so in rural areas. To address this gap, we performed a geographically detailed study of the rural food environment, summarizing and assessing various measures and identifying their association with a higher rate of diabetes and obesity.

**Methods:**

We surveyed 1,311 residents of Sullivan County, New York, collecting data on demographic characteristics and health status in January 2021. We assessed the food environment by cataloging restaurants and retail food stores and used least absolute shrinkage and selection operator (LASSO) regression to identify metrics strongly associated with diabetes and obesity (body mass index >30.0). We compared absolute proximity, density, relative proportions, and modified retail food environment index metrics. We applied fixed distance bands and nearest-neighbor approaches for proportions.

**Results:**

Older age (LASSO = +0.276), nearest-neighbor proportion of fast food restaurants (LASSO = +0.155), and low income (LASSO = +0.150) were associated with higher diabetes rates. Nearest-neighbor proportion (LASSO = +0.269), density per square mile of groceries/supermarkets (LASSO = +0.247), and Hispanic ethnicity (LASSO = +0.181) were the strongest predictors of obesity. Nearest-neighbor metrics helped with the problem of frequent zeros: the least skewed measures were fast food restaurants with 20 nearest neighbors (*P* = .37 for skewness) and counter-service restaurants with 20 nearest neighbors (*P* = .81).

**Conclusion:**

Nearest-neighbor approaches were less skewed to other metrics based on fixed distance bands, proximity, and density. Food environment metrics must be fully described and validated, especially in rural environments, before they are applied in research on critical health outcomes.

**Summary**

**What is already known on this topic?**

Although associations between unhealthy food environments and increased risk of diabetes or obesity have been studied extensively in urban areas, gaps remain in studying rural areas. One gap concerns the geographic metrics used to describe access in different food environments and how they perform in rural versus urban areas.

**What is added by this report?**

We performed a geographically detailed study of the rural food environment in Sullivan County, New York, summarizing and assessing various geographic metrics.

**What are the implications for public health practice?**

Geographic metrics that assess food environments must be fully described and validated, especially in rural environments, before they are applied in research on critical health outcomes.

## Introduction

Associations between unhealthy food environments and increased risk of diabetes or obesity have been studied extensively in urban areas but less so in rural areas of the US, where geographic differences in health outcomes continue to widen (1–5). Diabetes prevalence has been 1.2 times higher in rural areas than in urban areas, and death rates due to diabetes have been 1.7 times higher (1,2). Understanding modifiable drivers of this disproportionate burden of chronic disease on rural Americans requires precise measurement. These precise measures are needed not only because the distribution of food sources differ between urban and rural areas but also because studies that used the same metrics in urban and rural areas found different associations between the food environment and health outcomes in urban versus rural areas (6–9). These findings may be an artifact of how the food environment was measured rather than an actual difference in how the food environment affects urban and rural areas.

Robust analyses of how the food environment relates to rural health outcomes require accurate measurement of food sources at the correct geographic scale to determine how proximity to certain food sources can increase the risk of diabetes and obesity (10,11). Previous studies have measured food environments using various measures without considering whether they are properly applied in rural areas — whether they measure the food environment in a meaningful way (12–14). Because many studies used metrics that lacked proper validation (eg, description and summary of metrics before application, adjudication of whether the metrics relate to meaningful health outcomes) in rural food environments, the current literature is riddled with improperly performed studies that may have led to invalid conclusions about the influence of the rural food environment (15–18). Few studies have assessed how geographic measures of the food environment compare with one another in assessing health outcomes (13,19).

The objectives of this study were to 1) compare various measures of the food environment and how they operate in rural environments, and 2) analyze the association of rural measures of the food environment with diabetes and obesity.

## Methods

### Study design

We used a cross-sectional study design and data from a previous geographically detailed health survey of 1,311 residents in rural Sullivan Country, New York (20–22). The survey, mailed to all county households in January 2021, included questions that were consistent with previously validated national surveys assessing demographic characteristics and medical history such as the Behavioral Risk Factor Surveillance System (23–25).

### Study setting

Sullivan County is 61.5% rural according to US Census data in 2020 and has the third-worst county-level health outcomes in New York State; a high proportion of residents are non-Hispanic White, have low incomes, are in poor health, and have a high risk of developing chronic illnesses (26). This county was selected in a larger multipart study funded by the National Institutes of Health designed as a geographically precise examination of diabetes risk in rural areas that would also measure within-county differences in the rural food environment using data on residential addresses (22).

### Study participants

We obtained a comprehensive list of all households in Sullivan County from the Marketing Systems Group in October 2020 and pared down to all nonseasonal and nonvacant residential households that had a non–post-office box address. The list comprised 28,284 households, which was comparable to the 28,184 household indicated in 2019 US Census estimates (27). Survey respondents were offered a $10 gift card for participation. All survey responses were returned to the Sullivan County Public Health Department and then sent to the NYU School of Medicine, where response data were uploaded into REDCap (Research Electronic Data Capture) hosted by the NYU School of Medicine. REDCap is a secure, web-based application that supports data capture, collection, and management for research studies. This study was approved by the institutional review board at the NYU School of Medicine (study protocol s19–01920). All study participants provided informed written consent.

### Primary outcome

The main study outcome was the prevalence of diagnosed diabetes and high body mass index (BMI). To assess diagnosed diabetes, the survey asked participants if they had ever been told by a doctor or health professional that they had: 1) prediabetes, 2) gestational or pregnancy-related diabetes, or 3) diabetes. We categorized diabetes as type 1 or type 2 and gestational diabetes and prediabetes as “none.” We calculated BMI as weight in kilograms divided by height in meters squared based on self-reported height and weight; we defined high BMI as a BMI greater than or equal to 30.0 (ie, obesity).

### Health survey data

In January 2021, when health surveys were mailed, adult survey respondents aged 18 years or older were asked to provide information on their age, sex, race and ethnicity, and annual household income. Participants were also asked to indicate the number of adults and children in the household. Given the influx of people to Sullivan County during the summer, the survey asked participants if they were full-time or part-time residents. We excluded part-time residents from analysis. A full description of the study approach, survey questions and instrument used, and biases is available elsewhere (21,22).

### Food environment data

We assessed the restaurant and retail store food environments. We assessed the restaurant food environment by cataloging restaurants into fast food chains, counter-service restaurants, and wait-service restaurants. We categorized retail food stores as either unhealthy, which included convenience or dollar stores, or healthy, which included supermarkets or grocery stores. We made these categorizations by obtaining all available online data on each establishment. We examined online descriptions and photographs of each establishment to determine what food products or services they provided; when information was ambiguous, we contacted the establishment directly. For the restaurant food environment measures, we calculated absolute measures based on recognizable fast food restaurant chains or counter-service restaurants, which was done by dividing the number of fast food restaurants in the area by the total number of restaurants. The same was done for wait-service restaurants.

We used geospatial software to develop the various geographic measures of the food environment based on our review of the food environment literature (Table 1). We used absolute measures and relative measures (13,15,28–31). Absolute measures were distance from residential address to the nearest food source and density of stores per household or per square mile. Relative measures were the proportion of food sources within a given fixed-distance band (ie, 1, 5, 10 and 20 miles from residential address). We geocoded residential addresses to pinpoint the exact location of residence. We used the k-nearest neighbors algorithm (in other words, using the k-number of restaurants nearest to a study participant’s residential address) to develop a relative measure of the food environment that was independent of geographic scale, given the qualitative differences in absolute measures across the rural county. The number of nearest neighbors was specified as 5, 10, 20, or 30 neighbors to provide a range of measures. We restricted analysis to restaurants within 20 miles of the border of Sullivan County. 

**Table 1:** Measures of the Food Environment Included in Analyses in Sullivan County, New York, 2021

Methodology	Restaurant food environment	Retail food environment	Modified Retail Food Environment Index (mRFEI)
Equation used	• Relative measure: Unhealthy restaurants/Counter-service + Wait-service restaurants • Density measure: Unhealthy restaurants/Density units • Absolute measure: Distance to nearest fast food or counter-service restaurant	• Relative measure: Grocery stores + supermarkets/Grocery stores + supermarkets + Convenience stores • Density measure: Grocery stores + supermarkets/Density units • Absolute measure: Distance to nearest supermarket or grocery store	• Relative measure: Grocery stores + supermarkets/Grocery stores + supermarkets + Convenience stores + Counter-service restaurants
Approach and variation	• Unhealthy restaurants defined either as fast food restaurants or counter-service restaurants, including fast food • Distance band: Fixed distances within 1, 5, 10 and 20 miles • Density units calculated as either households within the distance band or the square miles within the distance band • Nearest neighbors calculated among 5, 10, 20, and 30 nearest neighboring locations	• Distance band: Fixed distances within 1, 5, 10 and 20 miles • Density units calculated as either households within the distance band or the square miles within the distance band • Nearest neighbors calculated among 5, 10, 20, and 30 nearest neighboring locations	• Distance band: Fixed distances within 1, 5, 10 and 20 miles • Nearest neighbors calculated among 5, 10, 20, and 30 nearest neighboring locations

This study evaluated only metrics that describe geographic accessibility; exposure to a particular food environment depends on many other factors, such as cost and marketing, which we did not measure. Being proximate to a particular environment does not necessitate a particular dietary behavior. This study sought to validate how these various geographic measures of the food environment operate in relation to cardiometabolic health outcomes among people living in rural areas (23,32,33).

### Statistical analysis

We summarized the demographic characteristics of survey participants and compared these characteristics with those from US Census Bureau’s 2021 American Community Survey 1-year estimates (34). Only 2% of survey participants had missing data on the main study outcomes (diagnosed diabetes and BMI); given this small percentage, we restricted the analysis to complete cases. We used least absolute shrinkage and selection operator (LASSO) regression to identify food environment factors strongly associated with diabetes and obesity (35–37). We compared absolute proximity distances, density measures, relative proportions, and a modified retail food environment index (mRFEI) metric, which is a blended measure of the retail and restaurant food environment, while controlling for individual-level predictors (age, sex, race and ethnicity, and income). We also performed tests of normality by assessing skewness of each metric for the rural food environment.

## Results

Study participants (N = 1,311) had a mean (SD) age of 59.5 (14.9) years. Most participants were non-Hispanic White (88.0%) and female (67.1%); annual household income levels were evenly distributed across 4 strata (Table 2). Compared with participants in the 2021 American Community Survey, our study participants were older and more frequently female and non-Hispanic White, but their annual household income distribution was similar. Of the study participants, 12.9% (n = 169) had diagnosed diabetes, with most (79.9%; 135 of 169) having type 2 diabetes. Mean (SD) BMI was 28.6 (6.4) excluding 2 participants who did not respond to this question; 34% (n = 445) of study participants were categorized as obese.

**Table 2:** Characteristics of the Study Population (N = 1,311) in Rural Sullivan County, New York, Compared With Characteristics in American Community Survey, 2021^a^

Characteristic	Study population, no. (%)	2021 American Community Survey, no. (%)^b^
**Age, y**		
18–39	308 (23.5)	19,710 (31.1)
40–59	258 (19.7)	23,005 (36.3)
60–79	386 (29.5)	17,365 (27.4)
≥80	359 (27.2)	3,232 (5.1)
**Sex**		
Female	879 (67.1)	31,878 (50.3)
Male	432 (32.9)	31,497 (49.7)
**Race and ethnicity**		
Hispanic	76 (5.8)	9,823 (15.5)
Non-Hispanic Asian	9 (0.7)	1,394 (2.2)
Non-Hispanic Black	46 (3.5)	5,070 (8.0)
Non-Hispanic White	1,153 (88.0)	44,236 (69.8)
Non-Hispanic Other	27 (2.1)	2,915 (4.6)
**Annual household income, $^c^**		
<25,000	298 (23.1)	14,069 (22.2)
25,000–49,999	281 (21.8)	14,957 (23.6)
50,000–99,999	395 (30.6)	16,351 (25.8)
≥100,000	315 (24.4)	18,062 (28.5)
**Diagnosed diabetes**		
Type 1	15 (1.1)	Not available
Type 2	135 (10.3)	Not available
Unknown type	19 (1.5)	Not available
None	1,142 (87.1)	Not available
**Obesity status**		
Obesity	445 (33.9)	Not available
No obesity	866 (66.1)	Not available
**Body mass index, mean (SD) [range]**	28.6 (6.4) [13.2–59.4]	Not available

a All values are number (percentage) unless otherwise indicated. Percentages may not add to 100 because of rounding.

b Ns vary depending on number of survey respondents.

c Twenty-two respondents did not provide a response; percentages are based on a denominator of 1,289.

### Primary analyses

#### Diabetes

The LASSO regression for diabetes risk showed a positive association with age (LASSO coefficient = +0.276, 32% increased odds) and the proportion of fast food restaurants within the 20 nearest neighbors (+0.155, 17% increased odds). Incomes from $25,000 to $49,999 (LASSO coefficient = +0.150, 16% increased odds) also showed a positive association, while non-Hispanic White race (LASSO coefficient = −0.092, 9% decreased odds) was negatively associated with diabetes risk. Density of grocery stores and supermarkets within 1 square mile showed a positive association as well (LASSO coefficient = +0.109, 1% increased odds). Higher densities of fast food restaurants within 10 miles (LASSO coefficient = +0.030, 3% increased odds) and non-Hispanic Black race (LASSO coefficient = +0.037) showed a slight increase in the odds of diabetes (4%), while annual household incomes of $100,000 or more (LASSO coefficient = −0.014) showed a slight decrease in the odds (1%) of diabetes.

#### BMI

The LASSO regression for BMI showed that the proportion of fast food restaurants using the 10 nearest neighbors had the strongest positive association (LASSO coefficient = +0.269) with 31% increased odds of obesity. The density of fast food restaurants within 5 miles (LASSO coefficient = +0.166, 18% increased odds), the proportion of counter-service restaurants within 1 mile (+0.033, 3% increased odds), density of grocery stores or supermarkets within 1 square mile (LASSO coefficient = +0.247, 28% increased odds), and Hispanic ethnicity (LASSO coefficient = +0.181, 20% increased odds) also showed a positive association with obesity. Asian race (LASSO coefficient = −0.134, 12% decreased odds) was a slight protective factor against obesity. The use of mRFEI was not associated with either health outcome.

### Food environment

**Figure 1:**
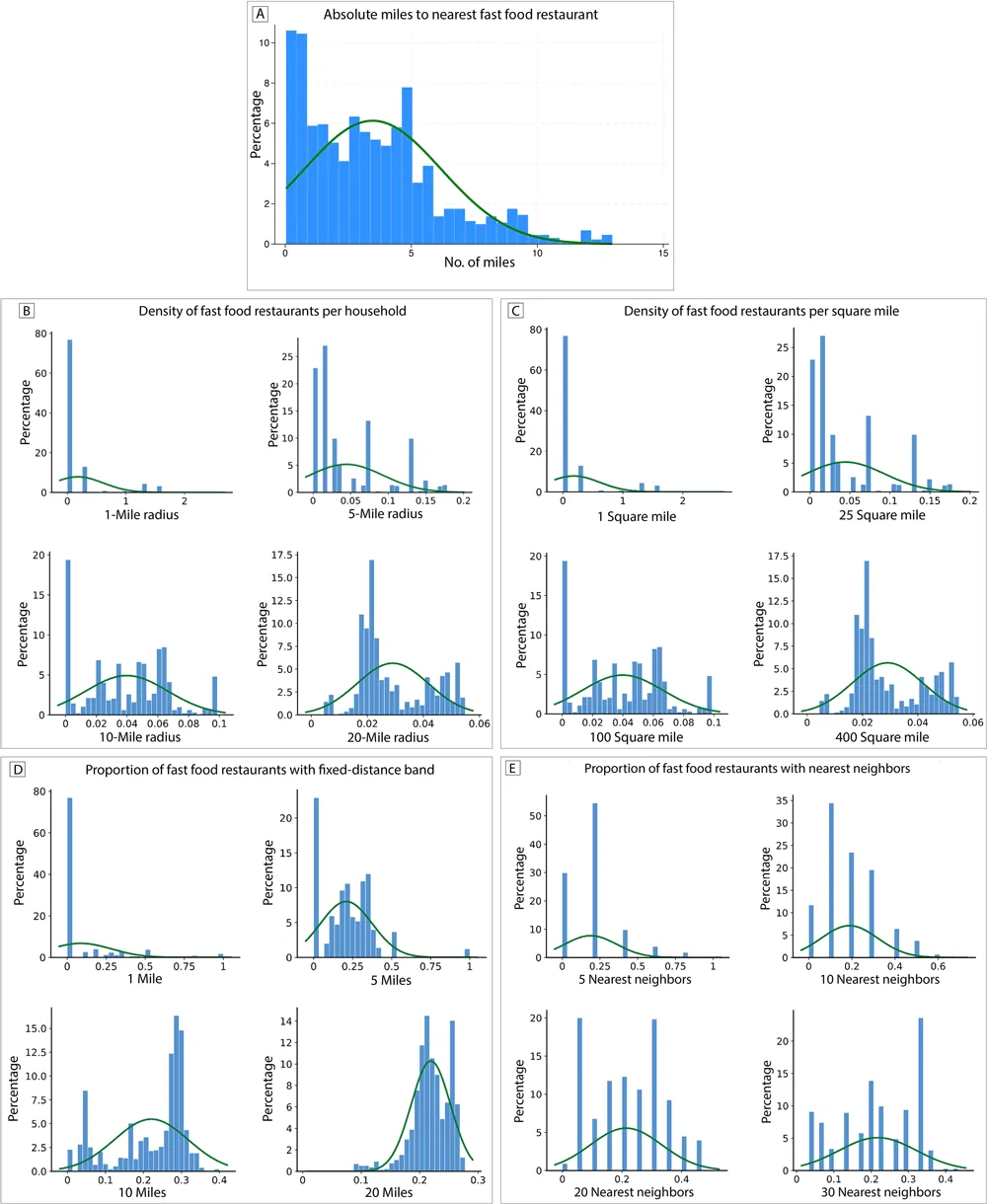
Histograms for various approaches to measuring the rural food environment for fast food restaurants in Sullivan County, New York, 2021. A) Percentage of households according to absolute number of miles to nearest fast food restaurant. B) Percentage of households according to density of fast food restaurants, by radius. C) Percentage of households according to density of fast food restaurants per square mile. D) Percentage of fast food restaurants within fixed distance band. E) Percentage of fast food restaurants with nearest neighbors. The green line indicates trend.

For the absolute distance to the nearest fast food restaurant, the measure was right-tailed, with most study participants being within 5 miles (Figure 1A); however, a small proportion of study participants resided more than 10 miles to the nearest fast food restaurant. The skew in density measures (fast food restaurants per household [Figure 1B] and per square mile [Figure 1C] within various fixed distance bands) was pronounced, except at higher fixed distance bands of 10 and 20 miles. However, because the county is only 33 miles at its widest point, the distance band of 20 miles is more than half of the width of the county.

We found that at the lowest fixed distance band of 1 mile, the proportion of fast food restaurants was also heavily right-tailed (Figure 1D), likely due to a high number of zero fast food restaurants in Sullivan County. This right-tail skewness began to even out as the size of the fixed distance band increased, switching to a left-tailed distribution by the largest fixed distance band of 20 miles. Overall, proportion measures showed skewness in both directions depending on the distance bands.

The proportion of fast food restaurants within k-nearest neighbors demonstrated a more even distribution of values among the study participants (Figure 1E). This even distribution was likely because these proportions accounted for differences in geographic scale. Therefore, the likelihood of zero values (due to no fast food restaurants being within range) decreased because the metric calculates the proportion of fast food restaurants among the nearest k-neighboring restaurants, regardless of whether the study participant lived in an area with a high or low density of restaurants. Nearest neighbor approaches showed little skewness and general evenness throughout all distance bands. The least skewed metric was 20 nearest-neighbors (*P* = .37 for skewness of fast food restaurants; *P* = .81 for skewness of counter-service restaurants) (Table 3).

**Table 3:** Results of Skewness Tests for Absolute, Density, Proportion, and Nearest-Neighbor Metrics in a Study on Measures of the Food Environment in Sullivan County, New York, 2021^a^

Measures	Probability of skewness
**Absolute, mi**	
Fast food restaurants	0
Counter-service restaurants	0
Grocery/supermarket	0
**Density per household **	
Fast food restaurants	
1 mi	0
5 mi	0
10 mi	0
20 mi	0
Counter-service restaurants	
1 mi	0
5 mi	0
10 mi	0
20 mi	0
Grocery/supermarket	
1 mi	0
5 mi	0
10 mi	0
20 mi	0
**Density (per square mile) **	
Fast food restaurants	
1 mi radius	0
5 mi radius	0
10 mi radius	.013
20 mi radius	0
Counter-service restaurants	
1 mi radius	0
5 mi radius	0
10 mi radius	.27
20 mi radius	0
Grocery/supermarket	
1 mi radius	0
5 mi radius	0
10 mi radius	.30
20 mi radius	0
**Proportion, mi**	
Fast food restaurants	
1 mi	0
5 mi	0
10 mi	0
20 mi	0
Counter-service restaurants	
1 mi	0
5 mi	.004
10 mi	0
20 mi	0
Grocery/supermarket	
1 mi	0
5 mi	.24
10 mi	0
20 mi	0
Modified retail food environment index	
1 mi	0
5 mi	0
10 mi	0
20 mi	0
**Proportion (nearest neighbor) **	
Fast food restaurants	
5 Nearest neighbors	0
10 Nearest neighbors	0
20 Nearest neighbors	.37
30 Nearest neighbors	0
Counter-service restaurants	
5 Nearest neighbors	0
10 Nearest neighbors	.15
20 Nearest neighbors	.81
30 Nearest neighbors	.006
Grocery/supermarket	
5 Nearest neighbors	0
10 Nearest neighbors	0
20 Nearest neighbors	0
30 Nearest neighbors	0
Modified retail food environment index	
5 Nearest neighbors	0
10 Nearest neighbors	0
20 Nearest neighbors	0
30 Nearest neighbors	0

a The probability of skewness test evaluates whether a variable’s distribution is significantly asymmetrical compared with a normal distribution.

**Figure 2:**
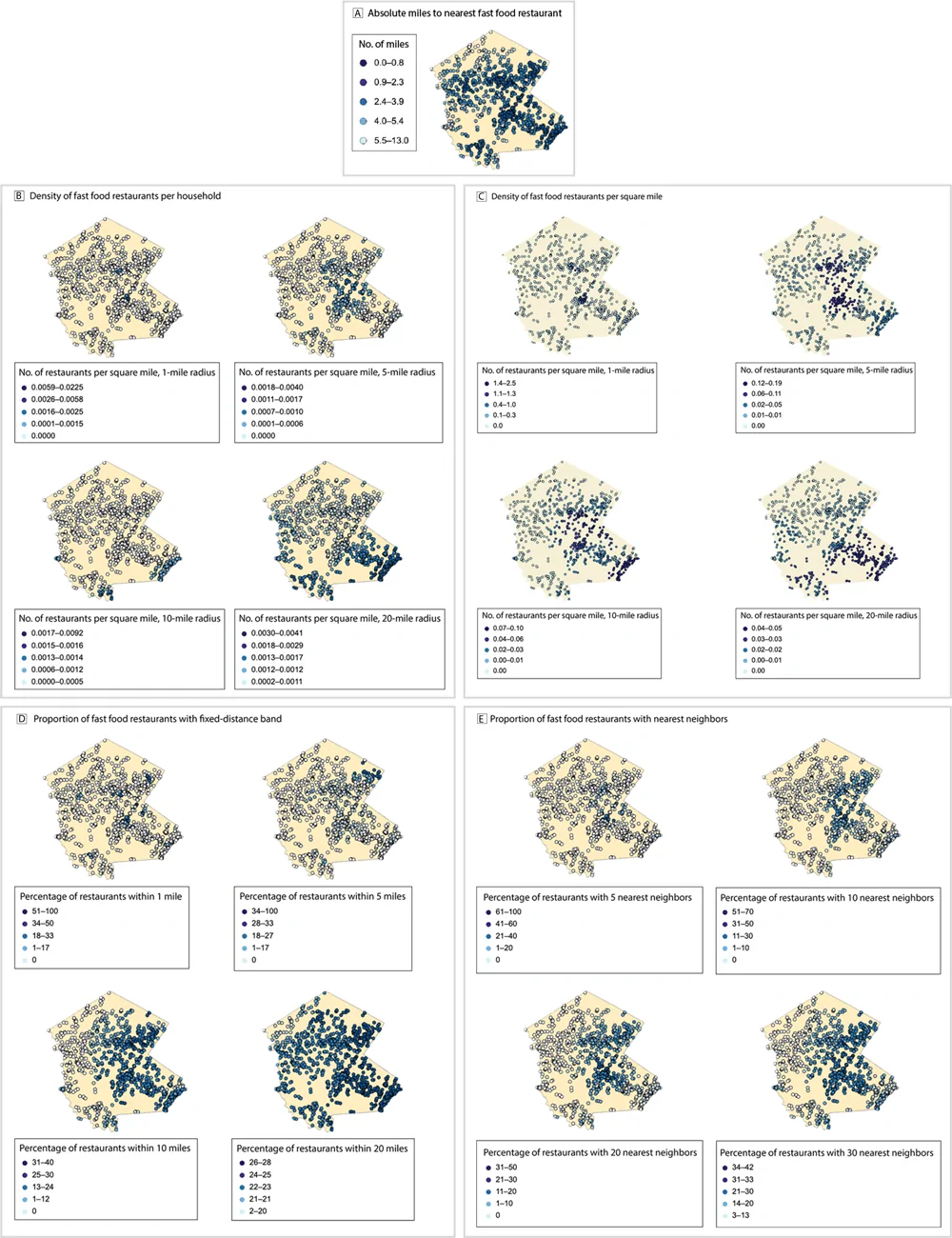
Maps for various approaches to measuring the rural food environment for fast food restaurants, Sullivan County, New York, 2021. A) Absolute number of miles to nearest fast food restaurant. B) Density of fast food restaurants per household. C) Density of fast food restaurants per square mile. D) Proportion of fast food restaurants within fixed distance band. E) Proportion of fast food restaurants with nearest neighbors.

Food environment measurements varied geographically across the county. The absolute distance to the nearest fast food restaurant followed a pattern related to the number of households in the area (Figure 2A). The density measures per household (Figure 2B) demonstrated particularly skewed results near the border of the county, which strongly suggests edge effects, especially at the larger fixed distance bands. We found a similar trend in density per square mile (Figure 2C), where it is particularly skewed near the border, further supporting edge effects at large, fixed distance bands. Relative measures (ie, the proportional measures of food sources) also demonstrated geographic patterns at low fixed distance bands that were similar to density measures at low fixed distance bands (Figure 2D). However, the use of k-nearest neighbors provided more stability in the geographic distribution of the food environment measure as the number of k-number of restaurants increased (Figure 2E).

In the analysis of grocery stores and supermarkets, we found considerable skewness in metrics based on absolute distance, density, and proportional measures. In contrast to our findings for the restaurant food environment, we found that measures with the least amount of skew for the retail food environment were the density of grocery and supermarkets per square mile using a 10-mile radius (*P* = .30) and the proportion of grocery and supermarkets within a 5-mile fixed distance band (*P* = .24) (Table 3). For the rural retail food environment, the high number of zeros in some metrics was still a problem, especially with smaller distance bands.

In addition, we found all proportions using mRFEI, regardless of approach (ie, fixed distance band or nearest neighbors), demonstrated significant skew (*P* > .001). The direction of this skew depended on the size of the fixed distance band or the number of nearest neighbors.

## Discussion

Although associations between unhealthy food environments and conditions such as diabetes and obesity have been extensively studied in urban areas, the same cannot be said for rural areas (33). Typically used measures of the food environment, which often describe urban food environments, do not operate well in rural environments. This study aimed to compare various assessments used to measure the rural food environment (23,32).

We compared absolute, density, and proportional food environment measures against one another and against our addition of k-nearest-neighbor–based metrics. While food environment measures using fixed distance bands can be useful in urban settings, issues may arise when applying fixed distance-bands to rural areas, where there is more variation in what 1 mile or 10 miles (which are the arbitrary fixed-distance bands used to measure food environment) means within a given rural area (38).

When assessing the restaurant food environment, absolute and density measures were highly prone to skewness and yielded results with large numbers of zeros, which may reduce their meaningfulness as a predictor. The results of the proportion measures also showed that when looking at great distance bands, the results tended to be more negatively skewed, and with shorter distance bands, the results were more positively skewed. Density measures tended to show substantial skew, especially for lower distance bands.

The k-nearest neighbors approach, however, accounts for geographic scale and aids in overcoming some of the challenges associated with analyzing the rural food environment. People living in rural communities often expect to drive longer to get to most places, so using the nearest neighbors approach to measure the rural food environment can better reflect the lived rural experience compared with proportion and density measures (39). Our results also supported the nearest-neighbors approach as being less skewed compared with the other measures. Using the nearest-neighbors approach to measure the rural restaurant food environment resulted in metrics that were the strongest food environment predictors for diabetes and obesity.

Determining the appropriate scale for food environment measures can be challenging because these measures may vary for different rural counties. Yet, overall, we found that most of the rural retail food environment measures were more skewed than the nearest-neighbor metrics. Both density per household and proportional measures using a fixed distance band showed left-skewed tendencies at small distances. The k-nearest neighbors approach, as applied to the retail food environment as it was to the restaurant environment, showed less skew and more consistency (ie, less left and right shifting in values with changing parameterization). An interesting finding for the rural retail food environment was that none of the metrics predicted diabetes or obesity except for the density of grocery and supermarkets within 1 square mile. In addition, the direction of the association between density of grocery and supermarkets per 1 square mile was positively correlated with diabetes and obesity.

This finding goes against the general literature, which is largely populated with studies performing county-level assessments of the food environment. It would suggest that the effect of density of grocery stores and supermarkets is different when comparing rural counties to each other versus residents within a given rural county. Our finding is consistent with our prior studies that found diabetes clustered close to rural towns, which might be due to socioeconomic factors that cause some rural residents to live in the more densely populated rural areas (40). Also, our finding that a high density of grocery stores and supermarkets within 1 mile was associated with diabetes and obesity may be confounded by other factors that determine where rural residents live and their risk of chronic disease.

When comparing different approaches for measuring the food environment in rural settings and their association with health outcomes, our nearest-neighbor approach was also more correlated with other metrics based on fixed distance bands, proximity, or density, providing further validation of its usefulness. In addition, we did not find that mRFEI metrics helped address skewness of metrics, nor did we find it to be associated with diabetes or obesity.

Unlike typically used measures, nearest neighbors can account for important features of the rural environment, especially as the travel required to get to healthy food outlets varies greatly across a rural county (40). This finding is important to consider when looking at previous studies of rural food environments that may not have considered issues of geographic scale. Also, if previous studies did not describe or validate the geographic measures before their incorporation into statistical analyses, then the results and conclusions of these studies might be completely invalid (41,42). We emphasize that we evaluated only geographic measures of the food environment; actual accessibility to certain foods depends on many other factors not measured in our study.

### Implications for policy and practice

Our study demonstrates the challenge in appropriately measuring the food environment in rural areas. While skewed metrics can still be informative, metrics with numerous zeros due to low population density or counts of food sources can render some calculations of food accessibility meaningless. More attention is needed to ensure that food environment measures are properly described and validated before their use in health studies. In addition, our study also suggests that the assessment of the food environment in rural areas needs to account for food sources often being located in rural population centers where diseases like diabetes tend to cluster. Therefore, our understanding of rural food access and association with disease needs to be measured at a more local level and account for within-county variation in the rural food environment.

### Limitations

As with any self-reported survey, the possibility exists of selection bias and errors in general self-recall. The study’s cross-sectional design also inherently precludes the ability to make causal conclusions; inferential methods may be needed to answer questions of causality. In addition, nonresponse bias to the brief health survey may have affected the associations identified in the study. In our prior analyses of the study population, we received responses from 8% of all adults in the rural county; however, respondents were more frequently older, female, White, insured by Medicare, or married without children (22). After raking procedures were applied, we found that the crude estimates of diabetes prevalence among survey respondents were higher than estimates that were adjusted to reflect the underlying distribution of residents based on census estimates (22).

An additional limitation is possible error in classifying food stores and restaurants. The study team individually assessed each establishment to determine its type. Given the small area of study, we preferred this approach to a standardized classification system, which may not have accurately reflected the characteristics of a given establishment. In addition, the associations we observed may have been confounded by other broader socioeconomic and geospatial factors such as income, real estate, degree of urbanicity, car ownership, or differences in the built environments. Finally, the mostly homogenous population of Sullivan County may limit the generalizability of these findings to other rural areas of the US.

### Future research directions

Future research should test the generalizability of our study findings in other rural areas of the US and investigate how the food environment should be measured in other areas of the world with the understanding that rural and urban areas may differ in geographic scale. Studies should consider the use of food environment metrics that are independent of geographic scale, such as the nearest-neighbor approach.

### Conclusions

This study demonstrated how various food environment measures operate against one another in a rural environment. Geographic measures cannot be applied in analyses without first describing them and assessing how they might operate in the region being studied. Nearest-neighbor metrics could be useful in assessing the rural food environment, and these assessments can guide local policies or actions to increase overall health and well-being in the area.
